# Broadly Neutralizing Antibodies against HIV-1 As a Novel Aspect of the Immune Response

**Published:** 2015

**Authors:** D. N. Shcherbakov, A. Y. Bakulina, L. I. Karpenko, A. A. Ilyichev

**Affiliations:** State research center of virology and biotechnology “Vector”, Koltsovo, 630559, Novosibirsk region, Russia; Altai State University, 61 Lenin St., 656049, Barnaul, Russia; Novosibirsk State University, 2 Pirogova St., 630090, Novosibirsk, Russia

**Keywords:** HIV-1, gp120, gp41, bNAbs, Broadly neutralizing antibodies

## Abstract

The human immunodeficiency virus-1 (HIV-1) has the ability to evade the adaptive immune response due to high mutation rates. Soon after the discovery of HIV-1, it was originally proposed that neutralizing of antibodies to the virus occurs rarely or cannot be elicited at all. In the 1990s, there appeared reports that sera of select HIV-1-infected individuals contained antibodies capable of neutralizing different virus subtypes. Such antibodies were named broadly neutralizing antibodies (bNAbs). Since 2009, the development of new cell technologies has intensified research efforts directed at identifying new bNAbs with a neutralization potency of over 90% of primary HIV-1 isolates. These antibodies have unique characteristics which include high levels of somatic mutations and unusually long variable loops that penetrate through the glycan shield of HIV-1 Env to contact the protein surface. In this review, we will attempt to summarize the latest data on bNAbs against HIV-1 in terms of their interactions with the sites of vulnerability on HIV-1 glycoproteins.

## INTRODUCTION

A distinctive hallmark of modern-day medicine in the last decade has been the increasing use of monoclonal antibodies offering targeted therapeutic effects for a range of disorders. A successful outcome with monoclonal treatment has been reported for dozens of commercial products over the past 15 years. Experimental data on the design and application of monoclonal antibodies have been reviewed in detail elsewhere [1, 2]. Although the mechanisms by which the humoral response is triggered and maintained remain elusive, new insight into broadly neutralizing HIV-1 antibodies (bNAbs) has expanded our understanding of the antibody response.

The human immunodeficiency virus type 1 (HIV-1), which causes the acquired immunodeficiency syndrome (AIDS), was discovered over 30 years ago. According to the WHO, > 78 million people were diagnosed as HIV-1 positive by the end of 2013, over half of whom have been reported dead. A safe and potent vaccine against HIV-1 could limit the spread of HIV-1 and subsequently eradicate the disease. The tendency of HIV-1 to rapidly accumulate mutations to escape host immune responses represents a major hurdle to the development of effective vaccines. HIV-1 has now been classified into 9 distinct subtypes and their recombinant forms [3].

**Fig. 1:**
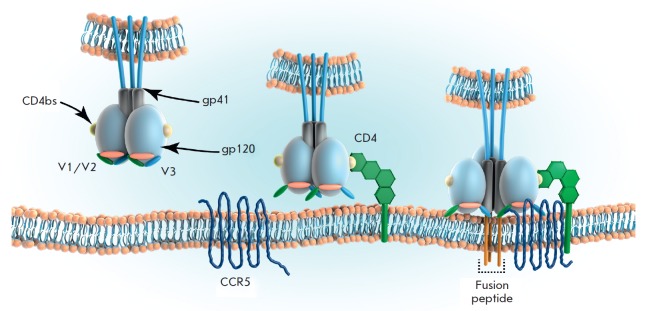
Trimeric Env interaction with the host cell membrane is illustrated. The gp120 subunit binds to the CD4 receptors, triggering conformational rearrangements to unmask the coreceptor binding site originally hidden by the V3 and V1/V2 loops. Engagement with CCR5 or the other coreceptor drives viral fusion and entry

Prior to 1990, it was considered that antibody-mediated neutralization of HIV-1 in the host was reduced or even abolished. In the 90s, it was found that sera of HIV-1-infected individuals contained antibodies that could recognize and neutralize different subtypes of HIV-1. These antibodies were called broadly neutralizing antibodies (bNAbs) [4]. Since 2009, with the advent of new cell-based assays, there has been a surge in the number of publications pertaining to the application of novel bNAbs. This review summarizes current literature on bNAbs, which suggests new possibilities for anti-HIV-1 vaccine design.

## STRUCTURAL AND FUNCTIONAL ORGANIZATION OF HIV-1 SURFACE GLYCOPROTEINS

HIV-1 is a spherical enveloped virus with a diameter of 140 nm. The viral envelope consists of a lipid bilayer derived from the plasma membrane of infected cells, with glycoprotein spikes anchored in it. Each viral spike is a trimeric heterodimer containing the external glycoprotein gp120 and the transmembrane glycoprotein gp41, with about 70–79 trimers on the virion surface [5]. Of all viral proteins, only gp120 and gp41 have epitopes for antibody recognition. These proteins play an essential role in virus entry into host cells.

The glycoproteins gp120 and gp41, which are encoded by the *env *gene, are called Env proteins and translate to a full-length gp160 polyprotein, followed by trimerization and cleavage by a furin-like protease in a Golgi compartment. The cleaved gp120–gp41 molecule is trapped in a metastable state until a transition to an energetically more favorable state. Like other Type 1 fusion proteins, these trimetric structures undergo receptor- induced conformational changes to increase the exposure of the gp 41 ectodomain for the fusion of viral and cellular membranes (*Fig. 1*). The crystallography on individual gp120 and gp41 components, as well as in the context of trimeric gp120/gp41, has been obtained in recent years, alongside mapping of gp120 CD4 and co-receptor binding sites [6].

HIV-1 infects cells through interaction with CD4 and chemokine receptors via transmembrane domains, such as CCR5 or CXCR4. Susceptible cells include T helper cells (Th), macrophages, follicular dendritic cells, Langerhans cells, and microglial cells. Certain CD4-negative cell types carrying chemokine receptors can also be infected. They include astrocytes, cervical cells, rectal and bowel mucosal cells, brain capillary and cervical endothelial cells, and corneal cells. CD4 serves as an adhesion molecule that stabilizes the viral contact with the host cell membrane [7]. The lack of attachment to the coreceptors prevents fusion from taking place; the virus enters by endocytosis and is typically inactivated upon uptake [7].

The ability of HIV-1 to rapidly accumulate mutations enhances the sequence variability of viral proteins. However, the domains within the proteins binding to CD4 and CCR5 are conserved. gp120 contains five conserved regions (C1-C5) that are interspersed between 5 variable regions (V1–V5). The variable loop regions occlude the constant regions to escape from antibody attack [8]. Following infection, antibodies are primarily raised against variable regions and, due to hipervariability, HIV-1 evades immune surveillance [9, 10]. Another mechanism by which the virus overcomes the immune defences is the glycosylation of surface proteins. It has been demonstrated that gp120 contains approximately 25 N-glycosylation sites, which form a glycan shield [8]. Mutations induce changes in the positioning of glycosylation sequences on gp120, thus altering the antigenic makeup of the viral envelope [11]. A virus mutant lacking certain variable loops and glycosylation sites becomes more susceptible to neutralization by polyclonal sera. This leads one to suggest that hypervariable loops mask the concerved epitopes of the Env protein [12]. With this in mind, it was a long-standing view that neutralization antibodies against Env antigens cannot be elicited in the course of the disease.

Indeed, knowledge on antibodies capable of neutralizing HIV-1 was lacking during the first years of HIV-1 research. Past evidence had posited that the human organism by itself was unable to limit viral replication, owing to its failure to raise neutralizing antibodies or, if mounted, their poor neutralizing capacity [18-20]. Recently, there have appeared reports on sera of HIV-1-infected individuals containing antibodies that neutralize both laboratory-adapted strains and primary isolates [21-26]. It was first suggested that broadly neutralizing HIV-1 antibodies (bNAbs) occur in a small proportion of HIV-1 infected patients [20, 27]. From then onwards, bNAbs were detected in some 30% of infected individuals diagnosed within one year of infection [23, 28, 29]. More recently, bNAbs have been found in over 50% of HIV-1 carriers [30]. Importantly, 1% of infected individuals elicit neutralizing antibodies with strong affinity for a wide array of primary HIV-1 isolates, as well as up to 99% of the HIV-1 isolates known to date [31].

Insights into bNAbs and their interaction with HIV- 1 could provide fundamental clues in our understanding of this phenomenon and may also be useful in the rational design of effective vaccine.

**Fig. 2:**
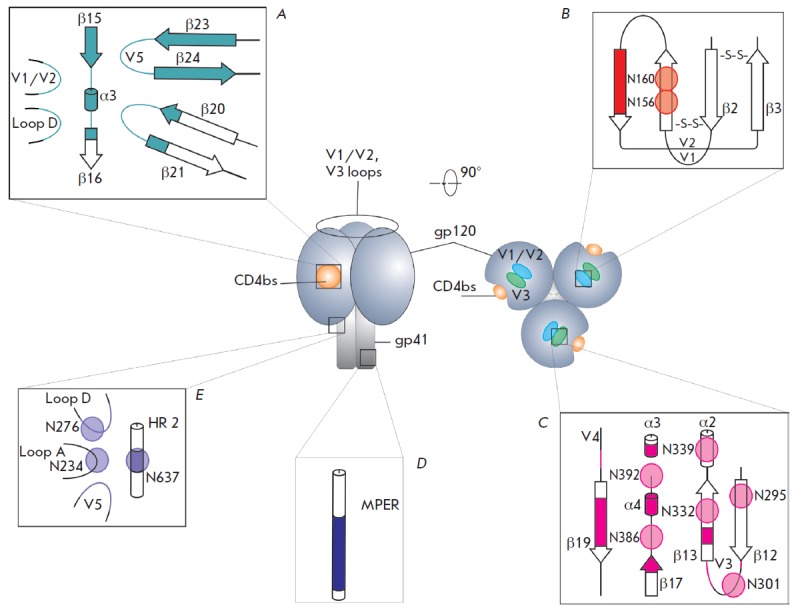
Schematic of the trimeric HIV-1 envelope glycoprotein structure and sites of vulnerability recognized by bNAbs are shown. The α-helixes are shown with cylinders, β-sheets, with arrows; loops, with thin lines; glycosylated amino acid residues, with circles. Detailed characteristics of bNAbs are given in Table. A – CD4bs on gp120 is involved in CD4 atachment. The major epitopes are the D loop, the V1/V2 loops, the V5 loops and the flanking β-sheets 23 and 24, an epitope within β-sheet 15, the α-helix3, and an epitope within β-sheet 16. The epitope structure is reconstituted based on data from [13]. B – the epitope made up of V1/V2. The antibody recognition site is a region in a β-conformation, including glycans at N156 and N160. The epitope structure is drawn based on Ref [14]. C – the epitope is on gp120. The sites involved in binding are: regions of β-sheets 19, 17, 13, V3, and V4 regions, 4 and 3 α-helixes, glycans at N392, N386, N339, N332, N301, and N295. The epitope structure is drawn based on Ref [15]. D – MPER-site, a linear epitope on gp41. A region within the MPER-site is amenable to recognition The epitope structure is drawn based on Ref [16]. E – the epitope at the gp120/ gp41 interface. N-linked glycans within gp41, a glycan moiety at N637, N276, and N234 V5 and D regions are targeted. The epitope structure is drawn based on Ref [17]

The HIV-1 trimeric complex gp41-gp120 has 5 sites of vulnerability to neutralizing bNAbs. Each site carries overlapping epitopes recognized by different bNAbs. These sites of vulnerability include the CD4 binding site (CD4bs) of gp120, the site within gp120 targeted by the PG9 and PG16 antibodies, and the membrane proximal external region (MPER) of gp41, an epitope adjacent to the V3 loop and spanning the gp120/gp41 interface. The five major sites of vulnerability are represented in *Fig. 2*.

Brief characteristics of bNAbs with the history of discovery and sites of binding are given in Table. The antibodies highlighted in grey are first-generation bNAbs.

**Table:** Characteristics of bNAbs against HIV-1

Envelope site	Epitope (specificity)	Antibody designation	Year of generation	Neutralization breadth, %	Neutralization potency*, μg/ml	The length of CDR H3, a.a.	Somatic mutations, %, aa substitutions
gp41 MPER	ELDKWA [18]	2F5**	1992	55–67 [39, 40, 57, 58, 59]	1.44 [40]	24	15.2
	WFD(I/L)(T/S) NW(L/I)WYIK [60]	4E10**	1994	85–100 [36, 39, 57, 58, 61, 62]	1.62 [40]	20	15.6
	SLWNWFDITN [63]	Z13**	2001	35 [62]	40 [62]	19	21
	WNWFDITN [63]	Z13e1**	2007	50 [36]			
	WFDITNWIWYIL/R [57]	10E8	2012	98–99 [40, 57, 58]	0.25 [40]	22	22.1
gp120 CD4bs	The loops D, V1/V2, V5 and CD4-binding loop	b12**	1991	35–75 [32, 38, 39, 61]	2.82 [39]	18	17.3
	The core epitope between the outer and inner domains, D474, M475 and R476 residues are important for recognition [64]	HJ16	2010	36 [61]	8.01	21	36.7
	The loops D, V1/V2, V5 and CD4-binding loop	VRC01	2010	88–93 [38, 40, 44, 45, 51, 57, 58, 65]	0.09 [45] 0.92 [48]	14	38.8
	The loops D, V1/V2, V5 and CD4-binding loop	VRC02	2010	90–91 [38, 40]	0.13 [40]	14	34.9
	The loops D, V1/V2, V5 and CD4-binding loop	VRC03	2010	51–59 [38, 40, 58]	0.08 [44]	16	34.9
	The loops D, V1/V2, V5 and CD4-binding loop	PGV04 (VRCPG04)	2011	77–88 [40, 44, 46, 51]	0.14 [40]	16	38.2
	The loops D, V1/V2, V5 and CD4-binding loop	CH31 (VRCCH31)	2011	84–91 [40, 44, 66]	0.02 [44]	15	31.9
	The loops D, V1/V2, V5 and CD4-binding loop	CH33 (VRCCH33)	2011	90 [44]	0.24 [44]	15	31.9
	The loops D, V1/V2, V5 and CD4	NIH45-46	2011	84–86 [40, 45, 48]	0.08 [45] 0.41 [48]	18	44
	The loops D, V1/V2, V5 and CD4	45-46^G54W^	2011	92 [48]	0.04 [48]	18	44
	The loops D, V1/V2, V5 and CD4	3BNC117	2011	86–92 [40, 45, 58]	0.06 [40]	12	36.9
	The loops D, V1/V2, V5 and CD4	12A12	2011	92–96 [40, 45]	0.07 [40]	15	34
	The loops D, V1/V2, V5 and CD4	VRC23	2013	65-80 [40, 58]	0.58 [40]	No data	No data
V1/V2 gp120 loop	Glycans at N160 and N156 and a β-sheet region within the V1/V2 loop	PG9	2009	77–83 [39, 40, 51, 57, 58]	0.08 [58]	30	15.4
		PG16	2009	73–79 [39, 40, 57, 58]	0.02 [57]	30	16.8
		PGT145	2011	78 [51]	0.29	33	22.8
		CH01	2011	46 [50]	3.75 [50]	24	23.3
gp120 V3 loop	Three glycans at N332, N339,N392	2G12	1994	28–39 [39, 40, 61]	1.45 [40]	16	33.6
	Complex-type N-glycans at N332 and V3 loop region	PGT121	2011	70 [51]	0.03	26	21.2
	High-mannose glycans and and β-sheet region at the C-end of the V3 loop	PGT128	2011	72 [51]	0.02	21	27.9
CD4i/V3 gp120	The V3 loop	3BC176	2012	64 [67]	12.8 [67]	19	29.4
Epitope at the gp120 / gp41 interface	Glycan-dependent epitope (a cluster of N-glycans terminated with a galactose residue at N611 and N637)	PGT151- 155	2014	64–66 [56]	0.008-0.012 [56]	28	No data
	gp120 D and V5 loops	8ANC195	2011	67 [45]	0.87 [45]	9	No data

Note. The rows shaded in grey are for first-generation bNAbs.

* - the percentage of neutralization is expressed as the amount of virus neutralized at IC50 valueslower than 50 μg/ml.

** – polyreactive.

## FIRST GENERATION bNAbs

The history of broadly neutralizing antibodies can be divided into two periods. The first studies of bNAbs appeared in the early 1990’s, reporting on b12, 2G12, 2F5, Z13, and 4E10 antibodies.

The first bNAb produced using phage display was b12, which binds to conserved gp120 CD4bs [4]. It was obtained as an antibody Fab-fragment generated from a phage display antibody library from the bone marrow of an HIV-1-infected non-progressor. The cloning of the variable region of the Ig heavy chain and light chain was random; therefore, such combinations may not occur naturally [32].

The 2G12 bNAb recognizes the α1→2 mannose residues on gp120, located close to the V3 and V4 loops [33], and has a unique structure. The heavy chains are intersected, with each light chain bound to the constant region of one heavy chain and the variable region of the other heavy chain. Due to this arrangement, Fab-fragments are unusually closely aligned. Such an antibody was obtained from only one donor library. The epitope recognized by 2G12 is conformationally sensitive, strongly depending on asparagine glycosylation in the C2-, C3-, C4-domains, and the V4 loop. ;

The 2F5 and 4E10 bNAbs interact with linear overlapping epitopes based around the MPER-region of gp41, exhibiting polyreactivity with bivalent heteroligation [34]. They have the ability to strongly bind to MPER with one Fab-fragment, while the other Fab-fragment demonstrates low affinity for another molecule target on the HIV-1 surface. The heterogeneous ligand binding seems to increase the neutralization activity against primary HIV-1 isolates [34].

Like b12, Z13 was generated from a combinatorial phage display library. To enhance affinity, amino acid substitutions were introduced to the paratope to generate a clonal variant, Z13e1, with a 35-fold increase in the binding capacity [19, 35, 36].

Studies carried out with HIV-1 pseudoviruses of different subtypes have demonstrated that first-generation bNAbs exhibit moderate breadth and neutralization potency. Achieving the desired efficacy against a wide range of HIV-1 isolates requires high concentrations of these bNAbs, which impedes progress in this field. At the same time, passive immunization of macaques with a combination of neutralizing Abs b12, 4E10, 2F5, and 2G12 confers complete protection against challenge by SHIV89.6P [37]. These findings spurred further studies aimed at identifying new bNAbs.

## SECOND GENERATION bNAbs

Numerous attempts to produce bNAbs with high, excellent characteristics had not met with success. The first bNAbs with enhanced efficacy and potency against a broad spectrum of primary HIV-1 isolates were only identified in 2009. The successful outcome was achieved through the use of three strategies: (i) Screening of sera from chronically infected HIV-1 individuals which contained high affinity and cross-reactive antibodies, (i) application of new approaches to B-cell selection and sorting, (iii) development of high-throughput procedures for generating human monoclonal antibodies.

The identification of VRC01 [38] and PG9/PG16 [39] was a breakthrough in the field of bNAbs in 2009–2010. The distinctive features of these antibodies are the strong neutralization profiles of a wide array of primary HIV-1 isolates and enhanced efficacy; 10-fold lower levels of antibodies are needed for protection with regard to first-generation bNAbs. VRC01, for example, shows neutralization activity of up to 93%; PG9/ PG16, up to 80% of primary HIV-1 isolates, whereas b12 (first-generation bNAbs) neutralizes only 35% [40].

VRC01 was produced using a novel strategy employed by Mascola *et al*. [38] that generated an antigenically resurfaced glycoprotein representing a substituted gp120 core, called the resurfaced stabilized core (RSC). To facilitate epitope selectivity using RSC as a probe, the CD4bs was preserved, the variable regions 1 to 3 removed, and other antigenic regions altered to reduce recognition. In addition, a ΔRSC probe with impaired b12 binding was used as a negative control. Sera containing NAbs to CD4bs were identified, followed by isolation of individual B-cells using the RSC and ΔRSC probes conjugated to fluorochromes. Single B-cells bound to the RSC probe were sorted. Single-cell RT-PCR [41] was applied to amplify cDNA encoding light and heavy chains of individual cells, followed by cloning into expression vectors that reconstituted the heavy- and light-chain constant regions [38].

PG9 and PG16 were identified earlier than VRC01, using a high-throughput strategy [39]. Activated B-cells were screened for antibodies with neutralizing activity against the primary HIV-1 isolates JR-CSF and SF162 and binding to the recombinant gp120 and gp41 proteins. The desired antibody genes were obtained from five B-cell clones. All five antibodies were tested for neutralization activity against a panel of pseudoviruses, and PG9 and PG16 demonstrating exceptional neutralization breadth and potency were selected.

## ENV SITES OF VULNERABILITY TARGETED BY bNAbs

**CD4 binding site**

Following the discovery of second-generation bNAbs, VRC01 enjoyed much attention due to its remarkable affinity for CD4bs of the HIV gp120 trimeric molecule. CD4bs, one of the prominent sites of vulnerability, harbors epitope for bNAbs (*Fig. 2A*). The existence of broadly reacting antibodies was hypothesized earlier [24, 42]. However, besides the monoclonal antibody b12, other broadly neutralizing antibodies escaped identification. Use of high-throughput strategies yielded three novel bNAbs (VRC01, VRC02, and VRC03) that recognize CD4bs. All of them were shown to be somatic variants with shared characteristics, with VRC03 displaying a limited neutralization breadth [38]. VRC01 has a number of distinctive features. First, it have a high level of somatic mutations in its variable regions. Somatic mutations usually account for 5–20% of VH genes, whereas VRC01 can carry up to 40%. Second, The variable domain of VRC01 is closely related in structure to the CD4 receptor on T helper cells. VRC01 displays structural mimicry of CD4 interaction with CD4bs on gp120 [13]. The precise targeting is a key determinant of high neutralization potency. Despite the close resemblance to the CD4 receptor, VRC01 interaction with gp120 considerably differs. Upon binding by CD4 to trimeric Env, the gp120 subunit undergoes structural conformations; by contrast, in the same context VRC01 traps gp120 in a state that prevents viral entry [43].

Following VRC01 identification, a myriad of CD4bs-binding antibodies, for example, PGV04, CH_3_0– 34 [44], 3BNC117, 3BNC60, 3BNC55, 12A21, 12A12, 8ANC195, 8ANC131, 8ANC134, NIH45-46, 1NC9, and 1B2530 [45], were obtained using the same strategy.

Notwithstanding that all these antibodies target a CD4bs epitope on gp120, considerable differences in the mode of action are observed [45]. For instance, certain antibodies bound to monomeric gp120 trigger conformational changes, reminiscent of those that take place upon binding by CD4, which is not shown for other antibodies [46]. Despite shared structural characteristics, CD4bs-binding antibodies could be encoded by different genes, allowing for a subclassification of VRC01-like antibodies [47].

Of particular interest is NIH45-46G54W, whose identification was made possible owing to the structure- based design based on NIH45-46, an antibody with exceptional potency and breadth against CD4bs. X-ray crystallographic data for the structure of NIH45-46 bound to gp120 revealed that a glycine to tryptophan substitution at position 54 increases the interactive surface between the antibody and the viral glycoprotein. Efforts to pursue the substitution yielded NIH45-46^G54W^, which displays enhanced potency and breadth [48].

**PG9 and PG16 recognition site**

PG9 and PG16 antibodies, which are somatic variants, show excellent neutralization breadth. PG9 neutralizes 78% of pseudoviruses; PG16, 73%. Importantly, the neutralization potency exhibited by both antibodies could vary by two orders of magnitude. These differ ences can be explained by the slight variation in the epitope binding sites recognized by PG9 and PG16.

**Fig. 3:**
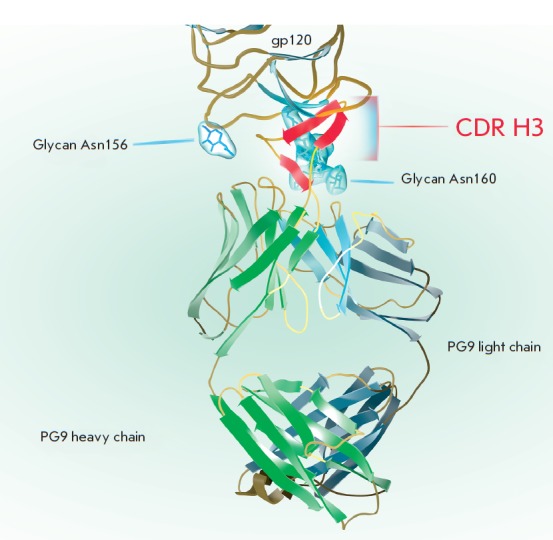
The PG9 Fab-fragment in complex with HIV-1 gp120 is shown. The variable and conserved domains of the heavy and light chains are in green and grey, respectively. The extended CDR H3 loop reaching through to the gp120 surface is highlighted. The β-sheets of CDR H3, critical for binding, are in red. The N-glycans at N160 and N156 through which the PG9 CDR H3 penetrates are shown as blue clouds. The schematic is reproduced based on the structures of 3U4E and 3DNN from Protein Data Bank

Glycosylation of N156 and N160 can affect PG9 and PG16 interaction with trimetic gp120. Unlike 2G12, whose binding requires glycans at N332, N339, and N392 [33], PG9 and PG16 involve both the N-glycosylation sites and amino acids of gp120 encompassed by the V2 and V3 loops [39]. Artificial proteins mimicking PG9 recognition in the gp120 context shed light on the structure of PG9 bound to the target. The CDR H3 loop of HIV broadly neutralizing antibody PG9 plays an essential role in stabilizing the PG9-gp120 complex. Its exceptionally long loop of 30 amino acid residues penetrates the glycan shield on gp120 to allow access to the protein surface around the V2 and V3 loops. The tip of the loop has a hammerhead structure, formed by two β-sheets. The outer β-sheet of the CDR H3 antibody makes four hydrogen bonds to the β-sheet at the base of the V2 loop (*Fig. 3*). Beside the hydrogen bonds, the negatively charged CDR H3 interaction with asparagine-linked sugar moieties at N160 and N156 also contributes to the interaction of PG9 with the epitope. The V3 loop and sugar moieties make up more than 50% of the contact-surface area [14]. This structure partially mimics the natural conformation of trimeric gp120 bound to the cell membrane [49].

PG9 and PG16 were among the first bNAbs used to target the second Env site of vulnerability spanning amino acid residues in the V1/V2 loops and oligomannose moieties at positions 160 and 156 (or 176) (*Fig. 2B*). Later on, CH01-04 [50] and PGT141-145 [51], which recognize the same epitope, were obtained [14]. The hallmark of these antibodies is their exceptionally long CDR H3, allowing penetration between the glycans and interaction with them, which contributes to enhanced binding to gp120 [14].

**V3 loop region**

The key residues of another site of vulnerability on HIV-1 Env are high-mannose glycans on N332 and a region within the V3 loop (*Fig. 2C*) [15]. The spectrum of antibodies recognizing this epitope includes the first-generation antibody 2G12, because the sugar moieties of gp120 that comprise the epitope 2G12 form a conformation similar to that of the 2G12 antibody. All other related bNAbs recognize not only carbohydrates, but also amino acids (*Table*). The structural shape of these antibodies and PG9-related antibodies allows them to recognize and pierce the gp120 glycan shield to interact with the protein surface beneath. PGT127-128 antibodies exhibit an elongated CDR H2 loop, and the PGT135 antibody has an extended CDR H1 loop [52].

PGT135 use long loops (CDR H1 and CDR H3) to penetrate the gp120 glycan shield, with CDR H3 playing the critical role. Beside the contact with the protein backbone of gp120, the flanking glycans also contribute to the interaction. CDR H3 interacts with glycans at N332, N386, and N392; CDR H1, only at N386. Like PG9, PGT135 and the carbohydrates of the epitope make up less than half of the overall interaction contacts which considerably contribute to the binding energy [15]. Sugar moieties play a minor role in recognition by another bNAb, PGT128, that interacts with glycan at N332, while N301 employs CDR H3 and CDR H2. The antibody’s elongated CDRH2 loop forms extensive interactions with gp120. Importantly, the moderate β-sheet at the tip of CDR H3 and the β- sheet structure of V3 on gp120 make hydrogen bonds critical for PGT128 recognition of Env [53].

Of note are PGT121 and 10-1074, which, in contrast to 2G12, PGT135, and PGT128, bind to complex-type N-glycans present in a low percentage on gp120, rather than high-mannose N-glycans [54].

If arranged in an order according to involvement of carbohydrates in the interaction, 2G12 ranks first for being fully carbohydrate-dependent, followed by PGT135, which is focused on binding to sugar moieties and to a lesser extent to amino acid residues, and PGT128, whose binding is strongly dependent on the CDR H3 contact with V3 amino acids. Interestingly, the neutralization breadth increases with the contribution of protein-protein interactions to epitope binding (*Table*). Following from this, the neutralization breadth seems to be driven by protein contribution to the contact surface. Although the glycan-recognizing antibody 2G12 can neutralize HIV-1 isolates, it demonstrates moderate breadth. By contrast, antibodies targeting the protein surface have increased breadth. The elicitation of glycan-recognizing bNAbs will likely exhibit poor potency.

**MPER region**

The fourth major site of vulnerability is gp41 MPER. Like CD4bs, it is very conserved and has been pursued as a target for bNAbs. MPER is critical for fusion and cell entry, hence MPER conservation is required to maintain its functions. Indeed, bNAbs, which recognize this target, were the first to be identified. However, sera of HIV-infected individuals that contained bNAbs against multiple HIV isolates showed that MPER-binding bNAbs are not common. In addition, the monoclonal MPER-directed antibodies 2F5, 4E10, and Z13 are polyreactive. At the same time, Huang *et al *(2012) reported a MPER-specific antibody, named 10E8 (*Table*), which neutralized ~98% of the HIV-1 isolates examined and did not show polyreactivity.

**gp120/gp41 interface region**

Broadly neutralizing antibodies which recognize both gp120 and gp41 have only recently been discovered [17, 55]. Like the majority of bNAbs (except for gp41 MPER specific antibodies), these antibodies bind Env through glycan-mediated interactions, but by contrast, critical glycan contacts are located on gp41. All the antibodies are structurally similar to other known bNAbs. 8ANC195 has a long CDR H3 loop and a protruding FWR3 (third framework region) of its heavy chain. This structure overcomes the glycan masking contributed by sugars at N234 and N276 to contact the gp120 D and V5 loops [17]. PGT151 has elongated CDR H3 and CDR L1 loops that are capable of recognizing Env in a conformation adopted prior to fusion of the viral membrane with the target cell membrane. Interestingly, PGT151-158-like antibodies can mediate antibody- dependent cellular cytotoxicity [56].

## CONCLUSIONS

The identification and investigation of bNAbs against HIV-1 has been a breakthrough in the understanding of the humoral immune response. Although these antibodies fail to prevent AIDS developing from HIV and virus clearance, bNAbs guide us through the remarkable adaptations of B-cell immunity in response to a sophisticated agent such as HIV-1.

Numerous studies of bNAbs have shown that the host’s immune system can accommodate HIV-1 escape mutations by generating unusual antibodies directed at hidden conserved epitopes.

The exceptional neutralization potency of bNAbs is mainly due to their structure. First, their hypervariable loops carry amino acid insertions in CDR loops, particularly CDR H3, which allow access to the gp120 protein surface through the glycan canopy. Second, bNAbs have the ability to accommodate epitope diversity by altering the conformation of their loops.

A distinctive feature of multiple bNAbs against HIV-1 is the engagement of the sugar moieties decorating the gp120/gp41 HIV-1 envelope glycoprotein. In addition to protein-protein interactions, carbohydrates effectively complement the antibody epitope recognition. Interestingly, glycan moieties could contribute equally or more than half to the overall binding energy.

Another important strategy is structural mimicry. There have been generated antibodies towards the site of CD4 attachment on HIV-1 gp120 that mimic important molecular details of the CD4-gp120 interaction.

These insights lend support, on one hand, to the potential of the immune system to address the challenge of pathogen diversity and, on the other hand, to vaccine design leading to the elicitation of potent bNAbs against HIV-1

## Abbreviations

HIV-1: human immunodeficiency virus type 1
AIDS: acquired immune deficiency syndrome
bNAbs: broadly neutralizing antibodies
Env: HIV-1 viral envelope protein
gp: glycoprotein
CD4: transmembrane glycoprotein receptor for an HIV-1
CD4bs: CD4 binding site
CCR5: C-C chemokine receptor type 5
CXCR4: C-X-C chemokine receptor type 4
MPER: membrane-proximal external region
RSC: Resurfaced Stabilized Core
RT-PCR: reverse transcription polymerase chain reaction
CDR: complementarity determining region
CDR H3: third complementarity-determining regions of the heavy chain
